# Feasibility study of a family- and school-based intervention for child behavior problems in Nepal

**DOI:** 10.1186/s13034-018-0226-3

**Published:** 2018-03-23

**Authors:** Ramesh P. Adhikari, Nawaraj Upadhaya, Emily N. Satinsky, Matthew D. Burkey, Brandon A. Kohrt, Mark J. D. Jordans

**Affiliations:** 1 Padma Kanya Multiple Campus, Tribhuvan University Kathmandu, Bagbazar, Kathmandu, Nepal; 2 Research Department, Transcultural Psychosocial Organization (TPO), Baluwatar, Kathmandu, Nepal; 3 Global Mental Health and Addiction Program, University of Maryland College Park, College Park, USA; 4 Johns Hopkins University, Baltimore, MD USA; 5 Department of Psychiatry and Behavioral Sciences, George Washington University, Washington, DC USA; 6 Centre for Global Mental Health, Institute of Psychiatry, Psychology & Neuroscience, King’s College London, London, UK

**Keywords:** Children, Behavior problems, School and family based intervention, Feasibility study, Psychosocial support, Nepal

## Abstract

**Background:**

This study evaluates the feasibility, acceptability, and outcomes of a combined school- and family-based intervention, delivered by psychosocial counselors, for children with behavior problems in rural Nepal.

**Methods:**

Forty-one children participated at baseline. Two students moved to another district, meaning 39 children, ages 6–15, participated at both baseline and follow-up. Pre-post evaluation was used to assess behavioral changes over a 4-month follow-up period (n = 39). The primary outcome measure was the Disruptive Behavior International Scale—Nepal version (DBIS-N). The secondary outcome scales included the Child Functional Impairment Scale and the Eyberg Child Behavior Inventory (ECBI). Twelve key informant interviews were conducted with community stakeholders, including teachers, parents, and community members, to assess stakeholders’ perceptions of the intervention.

**Results:**

The study found that children’s behavior problems as assessed on the DBIS-N were significantly lower at follow-up (M = 13.0, SD = 6.4) than at baseline (M = 20.5, SD = 3.8), p < 0.001, CI [5.57, 9.35]. Similarly, children’s ECBI Intensity scores were significantly lower at follow-up (M = 9.9, SD = 8.5) than at baseline (M = 14.8, SD = 7.7), p < 0.005, 95% CI [1.76, 8.14]. The intervention also significantly improved children’s daily functioning. Parents and teachers involved in the intervention found it acceptable and feasible for delivery to their children and students. Parents and teachers reported improved behaviors among children and the implementation of new behavior management techniques both at home and in the classroom.

**Conclusions:**

Significant change in child outcome measures in this uncontrolled evaluation, alongside qualitative findings suggesting feasibility and acceptability, support moving toward a controlled trial to determine effectiveness.

**Electronic supplementary material:**

The online version of this article (10.1186/s13034-018-0226-3) contains supplementary material, which is available to authorized users.

## Background

In low- and middle-income countries (LMICs), about 20% of children and adolescents suffer from mental illness [1]. Child behavior problems, including oppositional defiant disorder (ODD), conduct disorder (CD), and attention deficit-hyperactivity disorder (ADHD), are important to public health and human development as they are early indicators of later educational, social, emotional, and economic problems [2, 3]. Child behavior problems cause significant burden to families and societies through violence, disrupted relationships, and criminal acts [2]. Difficulties controlling impulses and behaviors often occur early in life [4], and commonly contribute to other mental health problems. These behavior problems comprise the major diagnostic risk factor for suicide [5]. Studies have shown that behavioral problems during childhood predict poorer social, educational, and economic outcomes as adults [6–9]. A meta-analysis of worldwide prevalence of ODD and CD showed similar incidence across geographic regions [10].

Behavior problems result from a complex interplay of biological, environmental, and experiential factors. Poverty, through exacerbating family dysfunction, has been associated with increased risk for CD and delinquency in children and adolescents [11, 12]. Exposure to violence, particularly frequent violent events, can also have adverse effects on children’s behavior, leading to school problems and an underdeveloped sense of right and wrong [13].

While Nepal’s economy rebounded during 2017, the South Asian country has been affected by a 10-year civil war, political uncertainty, and devastating earthquakes in 2015 [14]. The majority of the country’s population lives in rural areas and many of them experience mental health concerns [15]. Behavior problems have not been thoroughly assessed among children less than 18 years in Nepal. Previous research suggests that children with behavior problems in Nepal rarely seek or receive help [16, 17]. A study of physically disabled Nepali children found aggressive behavior (above the 98th percentile on the standard Child Behavior Check List (CBCL) criteria) in 12.5 percent of children [18]. Despite a need for programs to address behavior problems among children and adolescents in rural areas, mental health services in Nepal are concentrated in big cities [19].

Evidence suggests that behavior problems in children can be effectively addressed through parenting interventions. A systematic review of family and parenting interventions in high-income countries (HICs) found that positive effects can last through adolescence and into adulthood, as interventions reduced time spent in juvenile delinquent institutions and minimized re-arrest [20]. Similarly, a randomized controlled trial (RCT) of parent groups targeting child antisocial behavior demonstrated reduced ADHD symptoms in children [21]. While the majority of research on child behavior problems and the impact of treatments derives from HICs, recent interventions and evaluations have been performed in disadvantaged areas of HICs and in LMICs. Trials in LMICs have led to significant reductions in externalizing behaviors and adolescent risk-taking behaviors [22]. By providing parents with education, counselors are able to equip parents with skills to manage defiant behaviors and reduce rates of child non-compliance. Teaching parents pre-emptive strategies to address behavior problems, for example, has been shown to minimize children’s non-compliant behavior [23]. Parent Child Interaction Therapy (PCIT) in Puerto Rico boosted parent’s confidence in child behavior management and reduced impulsive, aggressive, and defiant behavioral patterns among children [3]. Another study, conducted in disadvantaged areas of the UK found that children’s behavior problems were significantly reduced at both 12 and 18 month follow-up assessments after a parenting intervention [11].

In addition to family-based programs, school-based interventions have been employed in LMICs to address child behavior problems. Studies have demonstrated mixed results. A school-based intervention in inner-city Kingston, Jamaica resulted in significant improvements in attendance and reductions in externalizing behaviors [24]; while a school-based intervention in Santiago, Chile failed to demonstrate a difference in mental health outcomes between the intervention and usual care groups [25]. A classroom-based psychosocial intervention in Nepal demonstrated reduced psychological difficulties and aggression among boys and increased prosocial behavior in girls [26].

Moreover, some literature suggests benefits of a multi-tiered approach where by intervention modalities are combined: generalized, school- or community-wide interventions with targeted components for high-risk individuals and their families [1, 27]. The present study aimed to evaluate the feasibility, acceptability, and outcomes of a combined school- and family-based intervention for child behavior problems in rural Nepal.

## Methods

### Identification of priority behavior problems

From 2013 to 2014, 72 free list interviews and 30 key informant interviews (KII) were conducted with community members of Chitwan District, Nepal, to assess parents’ and family members’ childcare customs and perceptions of child behavior problems [17, 28]. The interviews suggested a number of commonly experienced behavior problems among children in the community. The top five problems reported included; (1) addictive behavior, (2) not paying attention to studies, (3) getting angry easily and fighting over small issues, (4) disobedience, and (5) stealing [28]. Community informants suggested a combined school, family, and individual-based intervention to address the identified child behavior problems [16].

### Intervention selection and contextualization

**Fig. 1:**
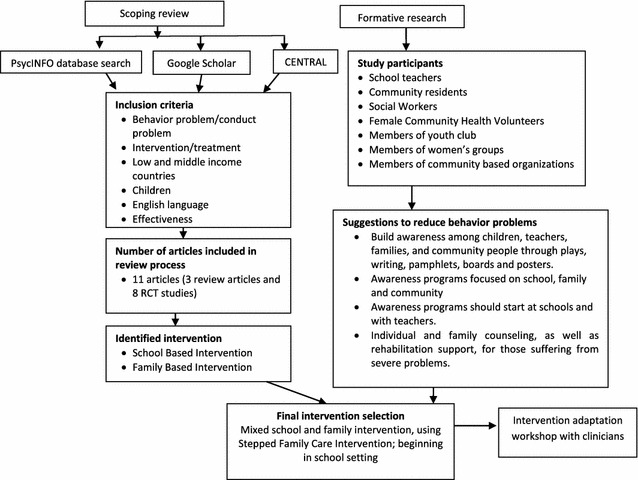
Selection process of intervention

To identify best practice in dealing with child behavior problems in LMICs, a scoping review was conducted using PsychINFO, CENTRAL, and Google Scholar. Altogether, eleven articles were identified. Three were review articles and the remaining eight were randomized control trials (RCTs) (Fig. 1). The findings of the review and results of the formative study guided the selection of the intervention, which was adapted for the Nepalese context through a workshop with Nepalese clinicians.

### Intervention adaptation workshop

**Fig. 2:**
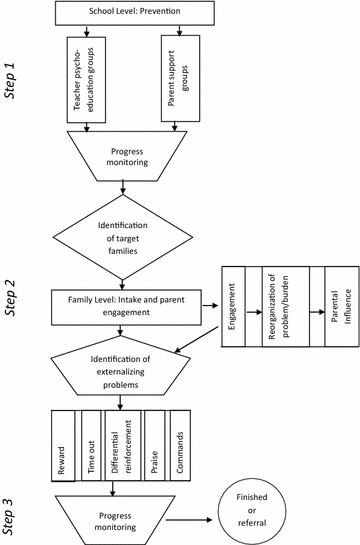
Description of intervention

The Stepped Care Family Intervention (SCFI) developed and implemented by Jordans et al. [29] was used as the basis for the family-based portion of the intervention. This tiered intervention was adapted for the Nepali context during a 1-day workshop at which psychosocial counselors, a teacher, a psychiatrist, and researchers collaborated to culturally adapt the intervention for use in rural Nepal. Altogether nine people with several years of experience in the field participated in the workshop. Based on the different intervention levels (school, family, and individual), three group discussions were established to discuss feasibility and acceptability. Following these discussions, the individual-focused level was removed, as participants agreed that it required substantial resources with only limited evidence for efficacy or potential for population-level impact (Fig. 2). The community-based intervention from the original SCFI was replaced with school based activities (for details see Additional file 1). Below we describe the adapted intervention in more detail.

#### Step 1: School level prevention

Psycho-education and awareness activities are provided for parents and teachers. The major objectives are to assess the externalizing behaviors and psychosocial problems displayed by children at school and in the household, and to teach parents and teachers how to deal with such behaviors. A psychosocial counselor conducts initial evaluations of the parent’s and teacher’s understanding of child behavior problems using emotion cards. During a group discussion, the counselor, teachers, and parents discuss major causes and impacts of these behaviors and current disciplinary practices. After the assessment, the psychosocial counselor provides psycho-education classes to groups based on identified needs. These classes include a brief introduction to child behavior problems, causes, impacts, and skills to effectively deal with specific behaviors (classroom management skills, student–teacher relationships, communication skills, rewards etc.).

#### Step 2: Family level intake and parent engagement

Family-level treatment is provided for children presenting with moderate-to-severe behavior problems. Trained psychosocial counselors work with parents to provide management strategies, enhance social support, improve family functioning, and reduce child behavior problems. The psychosocial counselors form parent support groups with parents of children with behavior problems. Based on geographic location, four to six parents are included in each support group. Psychosocial counselors facilitate a minimum of three group sessions and one follow-up session with each group. During these sessions, parents build social connectedness and support by sharing their stories, exchanging ideas, and exploring alternative ways of addressing family challenges and behavior problems.

#### Step 3: Progress monitoring

The counselors make home visits to assess the home environment and provide onsite support to both children and parents. Depending on the nature and severity of the child’s behavior problems, the counselors complete one to three home visits, during which the counselor works with the parents on behavior modification techniques: (1) training parents in a specific technique, (2) supervising implementation of the technique in the home setting, and (3) evaluating the impact of the technique. Techniques include: (a) selection of desired behaviors, (b) selection of reward system (chocolate or chewing gum, books, clothes, verbal reinforcement, cooking favorite food, physical affection), (c) using reward system *immediately* after desired behavior is shown, (d) explanation of reason for reward (labeling), and (e) consistency. To evaluate the impact of the technique, counselors use personalized outcome indicators based on which behaviors parents most want to see changed. These indicators are measured before and after the intervention. If low intensity care does not provide the expected gains (i.e. improvement in family functioning and reduction in the child’s behavior problems), counselors step-up to the next level of care. Stepping-up requires making decisions on the child’s progress based on judgments of ‘significant health gain’ or ‘improvement’ (for details see Additional file 1).

### Study setting and population

This study was conducted in the Meghauli Village Development Committee (VDC) of Chitwan District, Nepal. The study population consisted of children, parents, and teachers in the Meghauli VDC. After approval from the District Education Office and school principals, all teachers associated with government and private schools in the district were included. Self-referred parents of children ages 5–15 who voluntarily agreed to participate were also included. Although many children live in extended households with multiple adult figures, only parents were included. Children ages 5–15 with disruptive behaviors based on the Disruptive Behavior International Scale—Nepal version (DBIS-N) [30], and their parents were included if both children and parents provided consent.

Initially, psychosocial counselors provided 1 day of psycho-education on child behaviour problems to 201 teachers from 12 schools, and 100 parents, after which psychosocial counselors requested teachers and parents to refer children with behavior problems, based on judgement. Altogether, 104 children were referred. Using the DBIS-N, two researchers conducted screening interviews with parents of all 104 children. After screening, 41 children scored above the cutoff (≥ 17). All were included in the intervention after parents and children gave consent. At follow-up, 39 of the 41 children who participated at baseline were interviewed. The two children who did not participated moved to another district.

### Instruments

The baseline interview was conducted using the DBIS-N, the Child Functional Impairment Scale (CFIS), and the Eyberg Child Behavior Inventory (ECBI). After 1 week of the last intervention session, follow-up assessments were conducted using the same instruments.

#### Disruptive Behavior International Scale—Nepal version (DBIS-N)

The DBIS-N is a 20-item instrument which measures child behavior problems and which has been validated for use in rural Nepal. It includes 4 items assessing pro-social behaviors and 16 items assessing problem behaviors. The items are rated on a 0–3 scale based on frequency of occurrence (0 = “Never” to 3 = “Very Often”). Higher overall scores on the problem scale represent a greater number and/or frequency of behavior problems. The highest possible score for the DBIS problem subscale is 48 [30]. A score of 17 or above was used as the cutoff for inclusion, indicating moderate to severe behavior problems [31].

#### Child Functional Impairment Scale

Functional impairment was assessed using the CFIS, a tool that has previously been used in Nepal to assess a child’s ability to complete 11 routine daily functions expected of children in the study age range [32]. Each item is rated on a 0–3 scale with 0 representing no difficulty and 3 representing difficulty completing the task “most of the time”. Therefore, the range of potential scores on the CFIS is 0–33, with 33 representing the highest level of functional impairment across tasks.

#### Eyberg Child Behavior Inventory

The ECBI is a 36-item parent-report questionnaire that assesses child behavior problems using a 7-point scale to assess frequency and “yes/no” responses to assess the current presence of specific problems. The ECBI is scored according to “intensity” and “problem” domains, with “intensity” representing the summed numerical scores (range 36–252, where higher numbers indicates greater “intensity” of behavior problems) and “problem” representing the total number of items that are reported as being a “problem” for the informant (range 0–36, where higher numbers indicate a greater number of “problem” items) [33]. The ECBI was translated into Nepali by the authors of this study and approved by the authors of the ECBI.

### Implementation and supervision

Two counselors were mobilized for the three steps of intervention-delivery under the direct supervision of a clinical supervisor and the principal investigator (RPA). A clinical psychologist with knowledge of the intervention provided a 1-week training to both counselors. To further strengthen the quality of services and the uniformity of intervention delivery, the clinical supervisor visited the study community each week to provide supervision and feedback, with additional supervision via phone contact when necessary. Behavior changes were assessed at 4-month follow-up period.

### Qualitative methods

To assess stakeholders’ perceptions of the acceptability and feasibility of the intervention, a qualitative process evaluation was conducted. Using purposive sampling, a total of 12 people 4 teachers, 4 parents, and 4 community members participated in key informant interviews (KIIs) by the researcher. Semi-structured interviews explored stakeholder perceptions of the program, changes in children’s behavior, changes in behavior management, logistical concerns with the intervention, and recommendations for future delivery/scale-up of the intervention.

### Data collection

Two trained researchers with 2 years of experience in mental health research conducted the screening, baseline, and follow-up interviews. Both researchers received a 1-week training on the study objectives, design, overview of the intervention, ethics, and study instruments and semi-structured interview guide. At first, they conducted the screening interviews using the DBIS-N. If the screening instrument suggested that the children had behavior problems, they then conducted baseline interviews to collect household socio-economic information, the CFIS, and the ECBI. After the intervention, the same researchers conducted follow-up interviews.

### Data analysis

The quantitative data was entered into SPSS software and paired t-tests were conducted to assess differences in mean scores between pre- and post-intervention. Regression analyses were performed to explore predictors of child behavior problems. Thematic analyses were conducted with the qualitative data to establish themes on related topics. The collected qualitative data was first transcribed in the original language (Nepali) and then translated into English. After translation, the data was analyzed through creation of themes and subthemes.

## Results

### Background information

Of the total 41 children who participated at baseline, 31 (75.6%) were boys and 10 (24.4%) were girls. Participating children’s ages ranged from 6 to 15 years (mean = 10.7, SD = 2.8). Most children lived in nuclear families (65.9%) and a large proportion were from the Brahman/Chhetri caste (46.3%). Almost half of the children (41.7%) had fathers working in foreign employment. About two-thirds of the children (65.9%) had low food sufficiency status based on household production (Table 1).

**Table 1:** Socio-economic characteristics of study participants

	N	%
Age		
Less than 10	13	31.7
10–12	16	39.0
13–16	12	29.3
Total	41	100.0
Range and standard deviation	5–15 (2.8)	
Gender		
Girls	10	24.4
Boys	31	75.6
Total	41	100.0
Types of family		
Single parent	5	12.2
Nuclear	27	65.9
Extended	9	22.0
Total	41	100.0
Caste/ethnicity		
Brahman/Chhetri	19	46.3
Janajati	18	43.9
Dalit	4	9.8
Total	41	100.0
Father occupation		
Foreign employment	15	41.7
Daily wage labor	9	25.0
Service	7	19.4
Others (agriculture, business, self-employed)	5	13.9
Total	36	100.0
Sources of family income		
Own agriculture	4	9.8
Fieldwork for other landowner	4	9.8
Daily wage labor non-farming	6	14.6
Service	8	19.5
Foreign employment	16	39.0
Others	3	7.3
Total	41	100.0
Food sufficiency for the whole year		
Yes	14	34.1
No	27	65.9
Total	41	100.0

### Intervention outcomes

The paired sample t-test among the 39 children showed statistically significant reductions in mean DBIS-N problem scores, CFIS, and the ECBI. The change in the mean scores assessing impairment in daily functioning suggested that the intervention significantly improved children’s daily functioning. On average, the intervention reduced the DBIS-N score by 7.5, the CFIS score by 3.2, the ECBI problem score by 16.1, and the ECBI intensity score by 4.9 (Table 2).

**Table 2:** Comparisons of mean changes between baseline and follow-up (N = 39)

	Baseline Mean (SD)	Follow-up Mean (SD)	T (df); p	CI	% change
DBIS	20.5 (3.8)	13.0 (6.4)	8.0 (38); 0.000	5.57–9.35	− 36.6
CFIS	12.3 (6.1)	9.1 (5.6)	3.1(38); 0.003	1.13–5.23	− 26.0
ECBI problem score	107.9 (32.7)	91.7 (36.1)	3.2 (38); 0.003	5.84–26.41	− 15.0
Eyberg Intensity Scale	14.8 (7.7)	9.9 (8.5)	3.1 (38); 0.003	1.76–8.14	− 33.1

df, degrees of freedom; SD, standard deviation; CI, confidence interval

The intervention resulted in better outcomes in reducing DBIS-N scores among children from extended families compared to single parents, and among children from the Brahman/Chhetri caste compared to the Dalit caste. Likewise, the intervention resulted in a significantly larger reduction of the Eyberg problem score and intensity score in older children than in younger children, and in children from the Brahman/Chhetri caste than the Dalit caste. The intervention resulted in significantly larger improvements in daily functioning among children belonging to the Brahman/Chhetri caste compared with children from the Dalit caste (Table 3).

**Table 3:** Impact of the intervention by background characteristics (N = 39)

	DBIS		CFIS		ECBI problem score		Eyberg intensity scale	
	Beta (CI)	T (p)	Beta (CI)	T (p)	Beta (CI)	T (p)	Beta (CI)	T (p)
Time effects [baseline (ref.); end line]	− 8.0* (− 10.4 to − 5.6)	− 6.6 (0.000)	− 3.0* (5.6 to 0.4)	− 2.3 (0.02)	− 14.0* (− 28.1 to 0.1)	− 2.0 (0.05)	− 3.9* (− 7.3 to − 0.4)	− 2.2 (0.03)
Age	− 0.11 (− 0.6 to 0.4)	− 0.4 (0.67)	0.2 (− 0.3 to 0.7)	0.8 (0.44)	− 3.3* (− 6.2 to 0.3)	− 2.2 (0.03)	− 0.7* (− 1.4 to 0.1)	− 2.0 (0.05)
Gender [girl (ref.); boys]	2.3 (− 0.7 to 5.2)	1.5 (0.13)	2.3 (0.8 to 5.4)	1.5 (0.14)	2.2 (− 14.9 to 19.2)	0. 3 (0.80)	− 0.8 (− 5.0 to 3.3)	− 0.4 (0.69)
Types of home [single parent (ref.); Nuclear; Joint]	− 2.8* (− 5.9 to 0.3)	− 1.8 (0.07)	− 1.5 (− 4.8 to 1.7)	− 0.9 (0.36)	− 32.7* (− 50.8 to − 14.7)	− 3.6 (0.001)	− 2.4 (− 6.9 to 2.0)	− 1.1 (0.27)
Mother caste [Dalit (ref.); Janajati; Brahman/Chhetri]	− 3.3* (− 5.3 to − 1.3)	− 3.3 (0.002)	− 2.4* (− 4.5 to − 0.3)	− 2.3 (0.03)	− 23.2* (− 34.9 to − 11.5)	− 4.0 (0.000)	− 5.8* (− 8.7 to − 3.0)	− 4.1 (0.000)
Mother education [illiterate (ref.); primary; secondary; SLC and above]	− 0.4 (− 2.1 to 1.4)	− 0.4 (0.66)	− 1.2 (− 3.1 to 0.6)	− 1.3 (0.20)	2.9 (− 7.2 to 13.1)	0.6 (0.56)	0.5 (− 2.0 to 3.0)	0.4 (0.67)
Father occupation [foreign labor migration (ref.); labor; service; other]	1.0 (− 0.2 to 2.3)	1.6 (0.11)	0.1 (− 1.2 to 1.5)	0.2 (0.83)	4.6 (− 2.6 to 11.8)	1.3 (0.21)	− 0.1 (− 1.8 to 1.7)	− 0.1 (0.94)

CI, confidence interval

* Statistically significant at p < 0.05

### Perspective on parent management training

A mother of three children learned to replace her typical scolding and beating with loving and sweet words. Her youngest child, stubborn and disobedient before the intervention, showed behavioral improvements when the mother started asking him to do things from a closer distance (rather than yelling across a room), and by taking him gently by the hand. Instead of getting annoyed and impatient, she learned to show her child love and be more attentive in helping him study and read. She explained, “*If we bring changes in our behavior, we could also bring changes in their behavior.*” As the psychosocial counselors taught parents and teachers to demonstrate love and patience to the children, instead of instilling fear through beating and scolding, intervention participants saw tangible changes in children’s behaviors.

### Restructuring routines

In addition to changes in disciplinary practices, parents were also instructed in creating daily schedules so that their children could follow structured day-to-day routines. Many parents stressed behavior changes seen as a result of instilling routine into their child’s lives. Post-intervention, children more consistently washed, did homework, attended school, and ate meals in a scheduled manner. By allowing children to play after eating, instead of forcing them to immediately start work, parents noticed that their children demonstrated increased focus when it came time to study.

### Classroom changes

A teacher commented that instead of carrying a stick into the classroom, she started using inspirational methods to encourage students to work hard. She told her students: “*Whether you are here to play or to study, tomorrow you will need to be a doctor or an engineer*”. By giving examples of people in society who were on the wrong track because of poor habits developed early in life, she motivated her students to study and work hard. Another teacher explained that through a developed understanding of child psychology, teachers learned to create more favorable learning environments. They worked more closely with parents, let guardians know if there was a problem, and treated each child as an individual. Rather than using harsh techniques on the entire classroom, they made specific action plans to help struggling students. A high school teacher enacted a “No Punishment Zone” at his school, noting that the “*behavior of one teacher determines the future of the child*”. Following the intervention, if teachers beat their students they were liable to be punished, suggesting that the intervention led to sustained attitudinal and behavior change amongst teachers in the district. Teachers introduced new teaching methods and exercises to their classrooms based on psycho-education training. Before the intervention, some teachers had students copy answers even if children did not understand the questions—these teachers stopped this practice. One of the school principals started holding regular staff meetings to reiterate behavioral management techniques and to discuss challenges. During these meetings, teachers were encouraged to leave their stress at home and work toward a better understanding of child psychology.

### Child behavior problems

As a result of changes both at home and in the classroom, teachers, parents, the principal, and the counselor, saw reductions in child externalizing behaviors. A teacher noted that the children in his classroom “*used to have a 90% habit of getting angry, and now it [had] fallen to 60%*”. Other parents explained that their children started washing-up and studying without prompting. However, one mother noted that her child had reverted to his previous, disobedient state. She mentioned that children whose parents were not involved in the intervention were a bad influence on her son. While some children continued to lie and curse, all but one was significantly better behaved than before the intervention.

### Feasibility and community perceptions of intervention

Community informants were asked how community members perceived the intervention. The participants reported that community members generally appreciated the intervention. For example, one teacher said, *“when I talked with my students’ parents about the program, many laughed with joy as they were very pleased with the intervention*”. When asked whether the participants experienced any difficulties during the intervention, a few commented that they had difficulty attending meetings because of hectic work schedules. However, almost all informants mentioned that the counselors were flexible with their time and were willing to meet parents and teachers wherever and whenever was most convenient.

### Recommendations

Participants recommended that counselors work with more parents in the community. While the intervention primarily targeted parents of children presenting with behavior problems, participants reported that other parents may have similarly benefitted from psycho-education. Additionally, some informants suggested ongoing follow-up. For instance, one of the school principals explained that teachers would benefit from continued education and psychosocial support on child psychology and behavior. One of the intervention counselors mentioned that she and her team had to make adjustments to classroom management skills, teacher–student relationships, communication skills, and reward and reinforcement systems. This counselor suggested that future programs add more information on self-care. Extremely happy with the intervention, a school teacher advocated for more training sessions in order to include the entire village—parents, teachers, and students alike. While the Nepali conflict caused a huge economic and societal burden, he explained that “*this kind of program*,” can make society “*more effective, trustworthy, and fruitful*”.

## Discussion

This study examined the feasibility, acceptability, and outcomes of a stepped school- and family-based intervention for child behavior problems in rural Nepal. In both quantitative measures and qualitative reports, parents and teachers of children with behavior problems reported substantial improvements in children’s behaviors and functioning from baseline to follow-up. Parents and teachers both found the intervention feasible and acceptable to be implemented within a rural setting. Stakeholders in the community reported that the intervention brought important improvements in disciplinary practices both at home and at school. Improvements in behaviors at home were not isolated to participating families; rather, parents spread psycho-education to other community members, creating an environment supportive of positive behaviors among children and positive discipline and management among parents. Effectiveness studies assessing stepped family care models in India have shown similar findings; family-based interventions are appropriate even in poor and rural communities [34, 35]. This is consistent with the literature, including systematic reviews, observational studies, and randomized controlled trials, which suggests that positive parenting is a key factor in reducing child externalizing behaviors [11, 20, 36–39].

The mean score reductions on both the DBIS-N and the ECBI suggest significant improvements in children’s behavior problems. However, demonstration of effectiveness will require demonstration of statistical significance when compared with a control group. It is important to note that regression analysis suggested that the intervention was most effective among children belonging to extended families, among children from the Brahman/Chhetri caste, and among younger children.

Through the intervention, family members learned to deal with their children’s behavior problems through positive parenting and family adjustment. Family members were taught social learning techniques to improve children’s negative behaviors. The presence of multiple adults caring for children in extended families could potentially explain the greater reductions in negative behaviors seen among children in these groups, when compared to single-parent homes. In extended, or joint family systems in Nepal, several family members are responsible for caring for children and adolescents. Thus, having several adults engaged in positive parenting and family adjustment likely benefited children in extended families.

While school- and family-based interventions are often effective for low-income students with externalizing behaviors [40], class differences can impact effectiveness [41]. Children from the Brahman/Chhetri caste may have experienced increased reductions in externalizing behaviors compared to children from the Janajati and Dalit castes due to ingrained community- and self-stigma and caste-based discrimination against these groups [42]. Additionally, families from high castes, particularly those with intact family structures, are exposed to fewer effects of social determinants of mental health [43]. Children from lower castes are more likely to be marginalized, live in unstable family situations, and be exposed to poverty. In order to see similar reductions in behavior problems among the Janajati and Dalit castes, these groups may require additional social services.

Younger children may have seen more significant improvements in ECBI because of age differences in environment, brain development, and impulsivity. Older students likely spend more time away from the classroom and home environments. Thus, these students were less frequently exposed to teachers’ and parents’ new disciplinary practices and behavior management techniques. Furthermore, impulsivity increases dramatically during adolescence [44]. Due to limitations in brain development, adolescents are often unable to control this impulsivity [45]. This pattern may be stronger in emotionally reactive adolescents [45]. As the students involved in the present study demonstrated emotional reactivity, it is likely that older individuals demonstrated worse outcomes than their younger counterparts due to age discrepancies in brain development and impulsivity.

The pilot intervention had a number of strengths. The intervention was delivered by community psychosocial counselors who received an extensive, week-long training. Quality assurance was continually ensured through regular supervision by a clinical psychologist with knowledge of the intervention. The intervention was successful in mobilizing qualified psychosocial counselors. In future stepped-care implementation in Nepal, programs can maximize intervention reach (contact coverage) by employing community psychosocial workers. If strong support and supervision mechanisms are established, community psychosocial workers can more efficiently reach parents and teachers.

In addition to the strengths noted above, the intervention also had limitations. Due to a lack of control group, this study was unable to infer causality, and therefore determine effectiveness. Thus, this study was only able to assess feasibility and acceptability. Another limitation stemmed from the short follow-up period, as behaviors were only measured after 4 months. Future research should employ a longer follow-up period, whereby children’s behaviors are assessed on the three instruments at 6- and 12-month follow-up. Lastly, KII assessing stakeholders’ perceptions of the acceptability and feasibility of the intervention were overwhelmingly positive. These results could potentially be skewed due to social desirability bias.

As this study served as an initial feasibility test of the intervention, follow-up research employing an adequately powered sample size and a control group should be implemented to determine intervention effectiveness. If the intervention is deemed effective, future scaling-up of the intervention in surrounding VDCs should monitor and evaluate progress using larger sample sizes and assessing socioeconomic differences and other potential moderating factors more rigorously.

In future studies, parents of children without moderate-to-severe behavioral problems could be reached through further peer support, for example by training and supervising parents to lead parent peer-groups on Parent Management Training. By relieving the resources required by having psychosocial counselors or community psychosocial workers lead sessions, parent-led groups could give parents agency and provide more parents with necessary strategies in dealing with child behavioral problems.

## Conclusion

This study evaluated a stepped school- and family-based intervention for reducing child behavior problems in rural Nepal. The quantitative results demonstrated reductions in child externalizing behaviors, and parents and teachers involved in the intervention found the intervention acceptable and feasible for use with their children and students. Based upon the findings from this pilot testing, an RCT should be designed and implemented to determine the effectiveness of the intervention. If the intervention is shown to be effective for the Nepali setting, it should be further scale-up in surrounding VDCs and beyond to further reduce child externalizing behaviors, and subsequently, negative impacts at the family and community levels.

## Additional file

**Additional file 1.** Intervention X: Protocol.

## References

[1] Klasen H, Crombag A (2013). What works where? A systematic review of child and adolescent mental health interventions for low and middle income countries. Soc Psychiatry Psychiatr Epidemiol.

[2] Fergusson DM, Horwood LJ, Ridder EM (2005). Show me the child at seven: the consequences of conduct problems in childhood for psychosocial functioning in adulthood. J Child Psychol Psychiatry.

[3] Matos M, Bauermeister JJ, Bernal G (2009). Parent-Child Interaction Therapy for Puerto Rican preschool children with ADHD and behaviour problems: a pilot efficacy study. Parent Process..

[4] Kessler RC, Amminger GP, Aguilar-Gaxiola S, Alonso J, Lee S, Ustün TB (2007). Age of onset of mental disorders: a review of recent literature. Curr Opin Psychiatry.

[5] Nock MK, Borges G, Bromet EJ, Cha CB, Kessler RC, Lee S (2008). Suicide and suicidal behaviour. Epidemiol Rev.

[6] Loeber R, Dishion T (1983). Early predictors of male delinquency: a review. Psychol Bull.

[7] Kessler RC, Berglund P, Demler O, Jin R, Walters EE (2005). Lifetime prevalence and age-of-onset distributions of DSM-IV disorders in the National Comorbidity Survey Replication. Arch Gen Psychiatry.

[8] Barkley RA, Fischer M, Smallish L, Fletcher K (2006). Young adult outcome of hyperactive children: adaptive functioning in major life activities. J Am Acad Child Adolesc Psychiatry.

[9] Rey JM, Morris-Yates A, Singh M, Andrews G, Gavin W (1995). Continuities between psychiatric disorders in adolescents and personality disorders in young adults. Am J Psychiatry.

[10] Canino G, Polanczyk G, Bauermeister JJ, Rohde LA, Frick PJ (2010). Does the prevalence of CD and ODD vary across cultures?. Soc Psychiatry Psychiatr Epidemiol.

[11] Bywater T, Hutchings J, Daley D, Whitaker C, Yeo ST, Jones K (2009). Long-term effectiveness of a parenting intervention for children at risk of developing conduct disorder. Br J Psychiatry..

[12] Sheldrick CE (1995). Delinquency: risk factors and treatment interventions. Curr Opin Psychiatry.

[13] Wallen J, Rubin RH (1997). The role of the family in mediating the effects of community violence on children. Aggress Violent Behav.

[14] IFRC (2016). World disasters report.

[15] Luitel NP, Jordans MJD, Sapkota RP, Tol WA, Kohrt BA, Thapa SB (2013). Conflict and mental health: a cross-sectional epidemiological study in Nepal. Soc Psychiatry Psychiatr Epidemiol.

[16] Adhikari RP, Upadhaya N, Gurung D, Luitel NP, Burkey MD, Kohrt BA (2015). Perceived behavioural problems of school aged children in rural Nepal: a qualitative study. Child Adolesc Psychiatry Ment Health..

[17] Burkey MD, Ghimire L, Adhikari RP, Wissow LS, Jordans MJ, Kohrt BA (2016). The ecocultural context and child behavior problems: a qualitative analysis in rural Nepal. Soc Sci Med.

[18] Ojha SP, Khalid A, Nepal MK, Koirala NR, Regmi SK, Gurung CK (2000). An exploratory study of emotional and behavioural problems in physically disabled children and adolescents. J Inst Med..

[19] Luitel NP, Jordans MJ, Adhikari A, Upadhaya N, Hanlon C, Lund C (2015). Mental health care in Nepal: current situation and challenges for development of a district mental health care plan. Confl Health.

[20] Woolfenden S, Williams KJ, Peat J (2001). Family and parenting interventions in children and adolescents with conduct disorder and delinquency aged 10–17. Cochrane Database Syst Rev.

[21] Scott S, Sylva K, Doolan M, Price J, Jacobs B, Crook C (2010). Randomised controlled trial of parent groups for child antisocial behaviour targeting multiple risk factors: the SPOKES project. J Child Psychol Psychiatry.

[22] Klasen H, Crombag AC (2013). What works where? A systematic review of child and adolescent mental health interventions for low and middle income countries. Soc Psychiatry Psychiatr Epidemiol.

[23] Gardner FEM (1999). Parents anticipating misbehaviour: observational study of strategies parents use to prevent conflict with behaviour problem children. J Child Psychol Psychiatry.

[24] Baker-Henningham H, Scott S, Jones K, Walker S (2012). Reducing child conduct problems and promoting social skills in a middle-income country: cluster randomised controlled trial. Br J Psychiatry..

[25] Araya R, Fritsch R, Spears M (2013). School intervention to improve mental health of students in Santiago, Chile: a randomized clinical trial. JAMA Pediatr.

[26] Jordans MJD, Kamproe I, Tol W, Kohrt B, Luitel N, Macy R (2010). Evaluation of a classroom-based psychosocial intervention in conflict-affected Nepal: a cluster randomized controlled trial. J Child Psychol Psychiatry.

[27] Jordans MJD, Tol WA, Komproe IH, Susanty D, Vallipuram A, Ntamatumba P (2010). Development of a multi-layered psychosocial care system for children in areas of political violence. Int J Ment Health Syst.

[28] Adhikari RP, Upadhaya N, Gurung D, Luitel NP, Burkey MD, Kohrt BA (2015). Perceived behavioral problems of school aged children in rural Nepal: a qualitative study. Child Adolesc Psychiatry Ment Health.

[29] Jordans MJD, Tol WA, Komproe IH (2011). Mental health interventions for children in adversity: pilot-testing a research strategy for treatment selection in low-income settings. Soc Sci Med.

[30] Burkey MD, Ghimire L, Adhikari RP, Kohrt BA, Jordans MJD, Haroz EE (2016). Development process of an assessment tool for disruptive behavior problems in cross-cultural settings: the Disruptive Behavior International Scale—Nepal version (DBIS-N). Int J Cult Ment Health..

[31] Burkey MD, Adhikari RP, Ghimire L, Kohrt BA, Wissow LS, Luitel N, et al. Validity and psychometric properties of the Disruptive Behavior International Scale—Nepal Version: a scale developed using local stakeholder participation **(Draft submitted)**.

[32] Tol WA, Barbui C, Galappatti A, Silove D, Betancourt TS, Souza R (2011). Mental health and psychosocial support in humanitarian settings: linking practice and research. Lancet.

[33] Eyberg SM, Ross AW (1978). Assessment of child behavior problems: the validation of a new inventory. J Clin Child Psychol.

[34] Van Ginneken N, Maheedharia MS, Ghani S, Ramakrishna J, Raja A, Patel V (2017). Human resources and models of mental healthcare integration into primary and community care in India: case studies of 72 programmes. PLoS ONE.

[35] Patel VH, Kirkwood BR, Pednekar S, Araya R, King M, Chisholm D (2008). Improving the outcomes of primary care attenders with common mental disorders in developing countries: a cluster randomized controlled trial of a collaborative stepped care intervention in Goa, India. Trials.

[36] Gardner F, Hutchings J, Bywater T, Whitaker C (2010). Who benefits and how does it work? Moderators and mediators of outcomes in an effectiveness trial of a parenting intervention. J Clin Child Adolesc Psychol.

[37] Haggerty KP, McGlynn-Wright A, Klima T (2013). Promising parenting programmes for reducing adolescent problem behaviours. J Child Serv.

[38] Furlong M, McGilloway S, Bywater T, Hutchings J, Smith SM, Donnelly M (2012). Behavioural and cognitive-behavioural group based parenting programmes for early-onset conduct problems in children aged 3 to 12 years. Cochrane Database Syst Rev..

[39] Scott S, Knapp M, Henderson J, Maughan B (2001). Financial cost of social exclusion: follow up study of antisocial children into adulthood. BMJ.

[40] O’Donnell J, Hawkins JD, Catalano RF, Abbott RD, Day LE (1995). Preventing school failure, drug use, and delinquency among low-income children: long-term intervention in elementary schools. Am J Orthopsychiatry.

[41] Cutler D, Bulatao R, Anderson N (2004). Behavioral health interventions: what works and why?. Understanding racial and ethnic differences in health in late life: a research agenda.

[42] OHCHR. Shadow of caste and its stigma continue to violate all aspects of human rights. 2016. http://www.ohchr.org/EN/NewsEvents/Pages/Shadowofcaste.aspx. Accessed 15 Sept 2017.

[43] WHO (2014). Social determinants of mental health.

[44] Romer D (2010). Adolescent risk taking, impulsivity, and brain development: implications for prevention. Dev Psychobiol.

[45] Casey BJ, Jones RM, Hare TA (2008). The adolescent brain. Ann NY Acad Sci.

