# Supplementary material for: Feasibility study of a family- and school-based intervention for child behavior problems in Nepal
Source: Child Adolesc Psychiatry Ment Health. 2018 Mar 23;12:20. doi: 10.1186/s13034-018-0226-3 (PMC5865299; doi:10.1186/s13034-018-0226-3)
Supplement: Supplementary file 1 — Additional file 1. Intervention X: Protocol. [file 13034_2018_226_MOESM1_ESM.pdf]

SHARE/Nepal/July2014

Transcultural Psychosocial Organisation (TPO)
Nepal

Family- and school-based intervention for child behavior problems in Nepal

Brief Manual for counselors

TPO Nepal

INTERVENTION X: PROTOCOL

1. Introduction: How to help parents to support their children

Families in situations of conflict are put under more stress than in normal and healthy situations. War has negative effects on both individuals and social structures in society. Take for example the marks that violence leaves on families; family break-up, disharmony, changed family structures and increased parental substance abuse and domestic abuse can commonly be found. Due to the central role of the family in children's development and well-being, such negative effect vicariously impact children. At the same time families are the main system of protection, care and influence for children, and thus the most powerful in making sustainable changes with regard to the child's well-being. The parents are the natural child raisers, which means the parents' influence on the child can be used in psychosocial programming to deal with children's problems. Increasing their positive role in the child's upbringing in general, and in healing and recovery in specific, will strengthen them to cope with the situation and problems. Furthermore, the family might be, unknowingly, contributing to the problems experienced by the child. The experiences the parents have gone through, the parents psychosocial or psychiatric problems or the existing family patterns might all affect the child's well-being.

The family by far seems to be the most influential mediators of children's reactions to (non-familial) violence. The family's stability, safety, parental wellbeing end emotional sensitivity are often better predictors of social-emotional adjustment then exposure to violence *per se*. The role of the family in mediating such negative effects can be summarized as follows (Wallen & Rubin, 1997): (1) physical availability of the parents; (2) protection and physical safety by parental awareness about potential dangers and subsequently install rules, education and supervision; (3) support in working through traumatic events through communication and emotional sensitivity; (4) child rearing that fosters moral development to counter-balance the moral erosion as a result of conflict; (5) models of positive coping regarding safety, emotion regulation and sense of control.

Besides the family/parents, school is another important environment that influences the development of the child. In 2007 it was widely recognised – in Nepal – that corporal punishment was the norm in most schools, both public and private (Rising Nepal, 2007). Many factors contributed, such as teachers lacking training in child friendly discipline methods; lack of resources and facilities in the classrooms and schools, and overcrowding of classes. Together with a lack of accountability or regular supervision and support; it was easy for teachers to be stressed and frustrated, and use violence as a means of maintaining discipline and holding the attention of children (UNICEF, 2001). Parents may also believe that corporal punishment was necessary for their child's development and success, so children could experience violence at home as well as school (Ibid). The psychological effects of corporal punishment are more hidden but can include vulnerability to depression and a lowering of confidence.

Given the violence in Nepalese society within recent history (situation of conflict/war), it is imperative that in order to break intergenerational cycles of violence, corporal punishment needs to be eliminated from the schools and homes. There is growing evidence of the negative impact of corporal punishment and the social consequences of showing a child that violence is the way to handle conflicts and achieve one's aims. These consequences include increased family and gender-based violence, violent crime, and ultimately the propensity of a society to resort to violence-in acts of war, and acts of inter-communal violence (UNICEF, 2001).

As the situation of conflict/war influences individuals and social structures (like parents/ family/school/teachers), so does the family/school situation influence the development of the child. Sometimes the child may not present problems at home but may instead present behavioral problems at school (see figure 1).

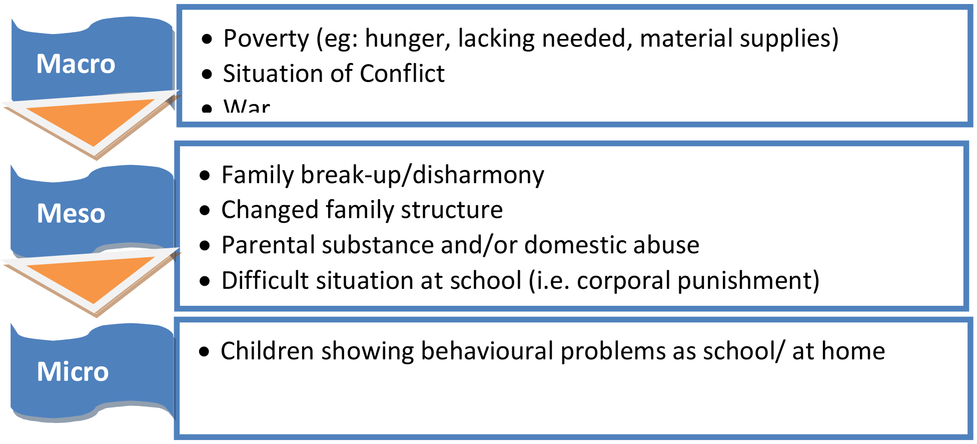

Figure 1: example how the different circumstances can influence people/children on different levels

When the child is showing difficulties at school, even simple school-based psycho-education and skills training can bring significant improvement to some traumatized children, while others might need more specific treatments and coping strategies (Klasen and Crombag, 2012). Another study demonstrated efficacy of the classroom-based-intervention (CBI) for improving social-behavioral and positive aspects of wellbeing indicators among subgroups of children exposed to armed conflict in a low-income country, Nepal (Jordans et al, 2010). Research has shown, low-cost, school-based interventions can substantially reduce child conduct problems and increase child social skills at home and at school (Henningham et al, 2012).

2. A stepped care model

Family therapies are a well-established method of treatment for children with psychosocial problems, with demonstrated effectiveness. However, issues of cost and efficiency in use of limited health care resources and professionals prevent large-scale provision of specialized therapies, globally but especially in low-income settings. Therapies provide poor access, with only minorities of people in need of care able to receive treatment (Bower, 2005). The use of stepped care models have been proposed to deal with the problem of access and equity. Stepped care basically means that care is divided in stages moving from lower-intensity and easy access interventions to higher-intensity and restricted access interventions based on the failure or lack of effect of the previous level of care. With this approach, more people receive care with the expectation that a significant portion of the people in need of care receive sufficient health gain through the lower level interventions and the higher level of care is reserved for those that need it most. A stepped care model means a combination of providing the least restrictive care (in terms of patient inconvenience, cost and service provider's time) and a self-correcting mechanism which means that the results of treatments and decisions about treatment provision are monitored systematically, and changes are made if current treatments are not achieving significant health gain ('stepping up') (Bower & Gilbody, 2005) – see figure 2.

Figure 2: Example stepped care model (adapted from Bower & Gilbody, 2005)

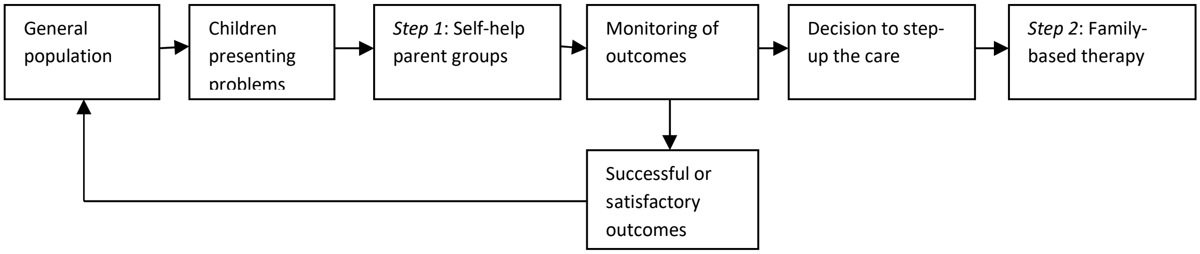

The stepped care model is based on the premise that even for the low-intensity interventions professional service providers are available, albeit in lesser service hours. Translating the stepped care model to a low-income setting with hardly any such professionals therefore entails reviewing the notions of least restrictive and specialized care. The different steps can be conceived as different levels of a public health approach, with the least restrictive level comprising of primary prevention intervention (largely community-focused) and the higher intensity level comprising of secondary prevention (focused interventions for individuals or groups). By utilizing a stepped care model, the interventions of the different levels are directly linked in one system of care with an internal screening and referral function.

3. Intervention modality

The family intervention described here follows the stepped care model. It has been developed through an intensive research-informed approach that entailed (a) an explorative study among stakeholders to identify perceived problems and the most pressing needs, (b) determining existing modalities of treatment for the targeted problems or population within other low- and middle- income countries through literature review, combined with priority-setting through an expert panel, (c) reviewing the literature for evidence-based treatments and subsequently distilling a profile of common practice elements among the reviewed efficacious treatments, and (d) assessment of feasibility of the treatment protocol through stakeholder consultation and consequently making adaptations to treatment content or modality to improve (cultural) acceptability.

This has resulted in a two-step intervention to support families with children presenting with behaviour problems. See figure 3.

Step 1: School-level prevention interventions
Step 2: Family-level treatment for children presenting with externalizing problems

Figure 3: Description of intervention

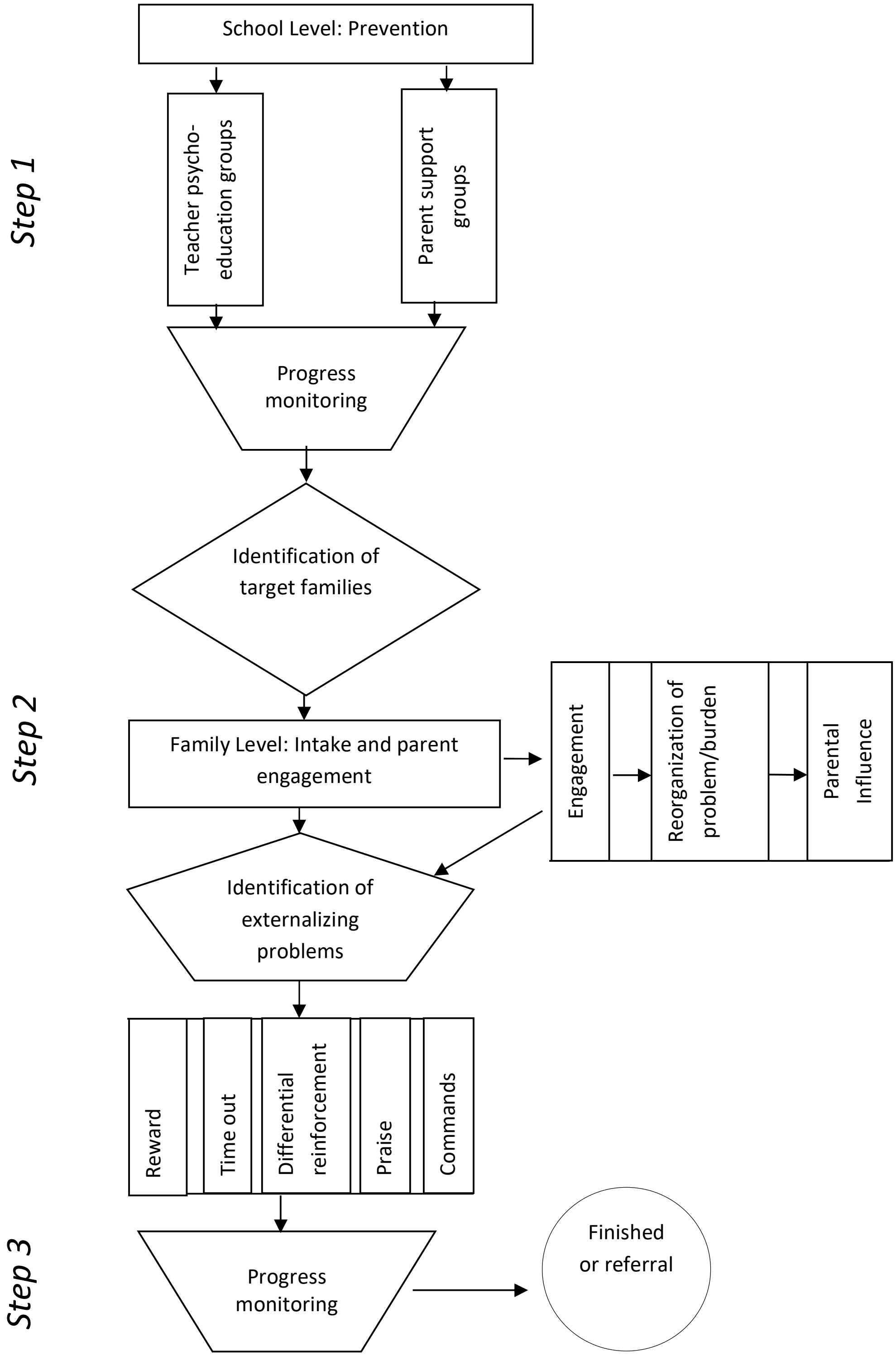

STEP 1: Prevention

PREREQUISITES:

- *The interventions described below should be part of a larger initiative that aims to improve the overall living conditions and well-being of the community at large. This entails community mobilization and primary prevention, such as school-based interventions targeting risk factors within the population, with the objective to increase community resilience, especially aimed at supporting the most at-risk families (e.g. poverty, marginalization, violence, substance abuse).*

The first step in the intervention protocol comprises the least restrictive, community- and school-level prevention interventions for the teachers, families and children. The overall aim of this level of care is to support teachers and families in dealing with children/adolescents presenting with, or at risk for, behaviour problems. The combined objectives of the step 1 interventions include:

- Support the teachers to learn how to identify and respond to children's psychosocial problems at school.
- Increase parental support, understanding and coping towards dealing with children's psychosocial problems.

Figure 4: Community and School-level

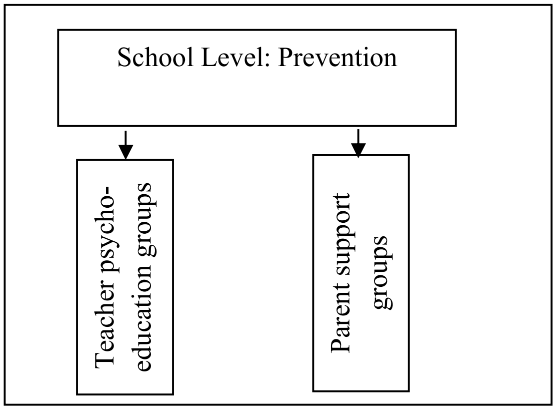

Preparation

Step 1 consists of three interventions that can be employed, together or separately depending on feasibility and need. Before any intervention can be implemented permission should be granted from relevant authorities and the interventions should be integrated, as much as possible, within existing care systems and school regulations. Counselors are trained to identify teachers and families in need of support in coping with the psychosocial problems of their children/pupils. Interventions, one or several, can be offered once indicated families/children are identified and their problems assessed to ensure that the intervention objectives match the needs of the family, teacher and child.

1.2 Teacher psycho-education group

Giving the teachers school-based psycho-education is an important intervention to raise awareness and to make sure the teachers can detect the psychosocial problems of children in the right way. It also contributes to improving their own skills in coping with the behavioral problems of children in the classroom. The school-based psycho-education for teachers, will give

them more insight into what kind of behavioral problems are common for children at a certain age and what psychosocial problems need attention. Recognizing psychosocial problems within children and possessing the skills to deal with these, serves an important preventive function.

a. Providing psycho-education in groups to teachers will raise awareness and create a better detection of worrisome behavioral problems. There will be two separate groups: one of the teachers of government school and next of the private school. Each group will have 10-12 teachers. Prompt detection at an early stage means children can be shown a different way of coping (with different outcomes). Also, these groups can be a platform for the teachers to exchange their sorrow/stress with each other.
b. Teaching the teachers different kinds of techniques and/or skills to manage the behavioral problems the children show in the classrooms

Step1: Teacher Psychoeducation group:

		Activities	Process	Methods
Step I	Day 1	Assessment	• Assessment of behavior problem of the children<br>• How they felt on the situation.<br>• How they responds on that behavior problem.<br>• Commitment for the continuation of teacher's meeting group	• Discussion
	Day 2	Provide psychoeducation	• Child development<br>• Skills to deal with specific problems (classroom management skills, teacher student relationship, communication skills, rewards, reinforcement etc.)	• Lecture and discussion<br>• Use audio/video<br>• Case discussion

1.3 Parent support groups

Support groups are facilitated group discussion to help people deal with a certain problem. Trained facilitators mediate the discussion and provide psycho-education where appropriate. Different support groups, or different sessions within support groups can have specific topics. The idea behind support groups, as the name suggest, is that members are able to provide support to each other, both through the realization that one is not alone in dealing with the problem of choice and through exchange of coping strategies (or resources) between members. This approach was deemed acceptable and feasible in Nepali culture. Furthermore, support groups provide an opportunity for social connectedness and support. Within the current intervention protocol there we distinguish two components of support groups:

a. Parenting support group that consist of sessions with mothers and/or fathers on caregiver-child interaction and coping with children's problems. These are essentially discussion groups with varying degrees of structure. Group members share their parenting difficulties and successes, parenting skills, and explore different ways of dealing with family challenges and everyday issues. See annex 1 and 2.

☆ Child rearing support means treading a sensitive balance between the parents' discretion regarding their methods and beliefs of child rearing and intervening when a child's

problem indicates such need. Discussion, practice and feedback are instrumental in making actual changes when needed.

Step1: parent support group

Before the formation of parent support group the following activities will be done:

- Identifying the family with children from the school
- Inviting parents for the workshop or training

		Activities	Process	Method
Step 1	Day 1	Awareness and Assessment	• Awareness raising and identification of behavioral problem in children<br>• Formation of parent support group of children having behavioral problem	• Discussion<br>• Case sharing
	Day 2<br>Day 2	Psychoeducation	• Psychoeducation on effective parenting and positive parenting<br>• Commitment of parents to attend the meeting after a month/two months for evaluation	• Lecture and discussion<br>• Use audio/video<br>• Case discussion

☆ Detection of children with behavioral problem needs to happen.

<u>Monitoring and evaluation</u>

As mentioned above the results of treatment should be monitored systematically. If the lower intensity care does not provide the expected gains (i.e. improvement in family functioning and reduction in the child's problems), stepping up to the next level of care is indicated. Stepping up requires a decision about patient progress based on judgments about 'significant health gain' or 'improvement'. Establishing such intervention impact might involve the use of personalized outcome indicators (or individualized expected outcomes). See annex 3.

➔ In case intervention gain is absent the family can be 'referred' to step 2 of the treatment protocol.

4.2 STEP 2: Treatment

PREREQUISITES:

- *The care components described in this section should be integrated within more generic counseling services. Counseling is defined as a planned and skilled interaction between the counselor and a client with the overall aim to improve the client's wellbeing. By listening attentively, and exploring difficulties or distress the client is experiencing, counseling provides an opportunity to work towards increased resources and capacity for overcoming difficulties. The counselors' role is to encourage expression of feeling and thoughts, transmit a sense of support and to facilitate resolution of problems. In general, counseling concentrates on the problems brought forwards by the client.*
- *Also, whenever possible, the treatment should be part of a larger care system that consists of more specialized mental health treatment for children or parents with severe psychopathology for referral and collaborative functions.*

The second step in the intervention protocol consists of family-level treatment for children presenting with moderate-to-severe externalizing behavior problems. Trained interventionists, for example counselors, work with individual families or parents, with the aim to improve family functioning and reduce the child's psychosocial problems. The specific treatment objectives are:

(a) Reduce children's externalizing symptoms.
(b) Improve parental skills to deal with children's difficulties.
(c) Improve family functioning through increased awareness on parental influence and strengthened parent-child relationships.

☆ As much as possible interventionists should follow the parent's perception of the child's problems. Home visitation should be encouraged as research has shown that this has a beneficial effect on parenting, improving assessment and treatment. Programmers and interventionists alike need to be sensitive to the potentially stigmatizing effect of problems and interventions.

IMPORTANT!

Before continuing with specific behavioral or emotional problems the counselors needs to assess whether there is a need for support regarding (other) ***basic child rearing, protection and development*** *issues. At times, parents may benefit from advice with regards to the children's basic needs (hygiene, good nutrition, sleeping habits, school work). The family who are facing the problems in the basic needs will be linked with the organization working in the sector. Similarly, the counselor will address and emphasize child protection issues. If there is any indication of neglect and abuse at this stage in the process, the counselor will need to engage in case-management, in order to mobilize support for the family to reduce potentially harmful situations within the family.*

1. *Basic child rearing advice and support*
2. *Case-management in case of potentially harmful situations*

2.1 Family intake

Figure 5: Family intake

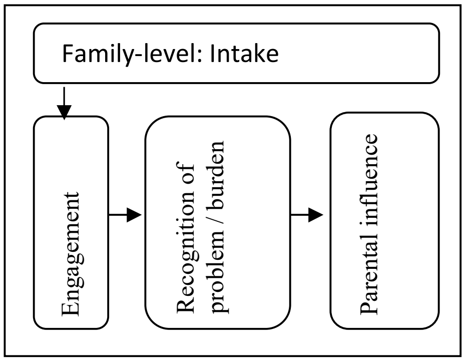

IMPORTANT!
It is important to remember that the primary mission of an initial family interview is to build a trusting, collaborative relationship with the family and the individual family members. Gathering information is secondary. If relationship-building is going slowly, it is fine to spread this interview over more than one meeting. One moves at the pace of comfort for the family to the extent possible. Another way to say this is: The purpose of the first interview is to be able to have a second interview. The ultimate goal is to create a comfortable shared relationship.

Pre-Engagement

Pre-engagement information and communication is very important between the family and the counselor. Crucial issues to consider here are:

a. *How has the family come to the attention of the therapist* (the referral process)?

For the referral process; did this family request to see a counselor? Was the family identified through a community screening? Does the family recognize a problem or is the indicator of the problem a high score on a screening instrument?, Is the family willing to meet with a counselor?, Is the family familiar with the concept of counseling or has counseling been explained to them?, Are there any known conflicts in the family about receiving help (e.g. mother wants to get help, but father does not)?, and Is the child with a behavioral problem already receiving care? All of these questions influence how to approach the first engagement.

b. *Minimizing potential barriers to engagement* (issues of location, gender and other social identity markers)

For potential barriers to engagement, issues of location, gender and ethnicity/caste should be considered. If the first family engagement is going to be at school, issues of gender and ethnicity/caste are minimized. However, if there is going to be a home visit, this could be difficult with a male counselor who is visiting a family with all females. For some families, they may be uncomfortable based on caste/ethnic differences if there is a home visit. Moreover, families may feel shame or stigma about having a counselor come to their home, and thus they may prefer to come to school.

How to address family members?
1. People can be addressed by their family and social position: dai, bhaai, didi, bahini, aamaa, buwa, etc.
2. It is appropriate to talk to the oldest person, usually the eldest man first. This may be the father in the household, or the eldest son. After addressing the person in authority, etiquette should be followed to greet others, so that they feel included in the conversation.

Engagement

A first meeting between counselor and parents should focus on introductions and explanations of treatment. The aim is to arrive at ***parental commitment*** to engage in treatment. Parents may not be aware of the problems of their children, or more commonly, of the impact of violent events on the child's well-being. The first steps are to create parental awareness and sensitivity about the identified problems and treatment, as well as commitment to cooperate to support the child.

a. *Rapport building*
   - Introduce yourself and the organization you're working for.
   - Because most families will be unfamiliar with the idea of mental illness, psychosocial care, and counseling, it will be important to explain counseling after your introduction
   - Explain about the **confidentiality**: It is crucial to discuss confidentiality during the introduction. The family should be aware that all of the conversation is between the family and counselor.

b. *Discuss aims for meeting/intervention (i.e. reduce problems of the child) and parental willingness to continue*
   - When meeting a family it is important to include an explanation by the clinician why the meeting is occurring and what is hoped to be accomplished. This explanation helps clarify the purpose of the meeting, as some family members may be puzzled that the whole family would be asked to attend, when only the child has behavioral problems. The meeting should convey respect and invite family members to participate collaboratively with the clinician.

c. *Explain intervention and research process*
   - The duration of the session(s)
   - The initial idea of the amount of sessions for the treatment
   - An agenda is made for every individual session (what to discuss in that session)
   - Short explanation about the techniques/process that we are going to use in the treatment

Mutual recognition of problem and parental burden

After the introductory stage, the service provider and parents do an assessment, to come to a mutual problem understanding. Service providers should thereby acknowledge and give attention to the difficulties the parents are experiencing in dealing with the problems in the family (recognizing parental burden).

Prior to talking about the understanding of the problem (1) and recognizing the parental burden (2), it is important ***to set some rules and guidelines***. Setting rules for the whole family in the treatment can also be experienced as a training session for the parents. In this way they see an example of the counselor, setting and following rules. This is a technique the parents later learn themselves, to assist in changing the problematic behavior of their child(ren).

Important rules and guidelines

- One person speaks at a time and do not interrupt until the person is done speaking.
- There is no right or wrong answer, everyone's opinions are important and should be respected.
- Use appropriate body language to show respect for whom ever is speaking.
- Everyone has a role in making the problem in the family better, so everyone should share during the family engagement. Everyone's voice is important. Everyone has a responsibility.

a. Mutual problem understanding (see also annex 4)

A more in-depth understanding of the child's problems and reasons for these problems will be the basis for continuing the intervention to maximize the fit between problems and treatment. The importance of this stage is to respect the perspective of the parents, to create awareness on the problem and to involve them in developing problem statements that includes their vision and needs. The assessment should further determine the treatment trajectory.

It is vital that the service provider learns what family members believe about the causation, maintenance and the expected course of the problem, as well as their beliefs about what treatments or changes are needed for it to remit and any concerns about involving a mental health clinician in addressing it. Questions can be asked such as:

"You have lived with this problem for some time, so it is important that I hear what you think about it. What have different family members believed about:

1) *What caused the problem?*
2) *How serious do you believe it to be? "What consequences could occur as a result of these problems?*
3) *What course do you expect it to take if it continues over time? "How long will it last?" "Will it get better on its own?"*
4) *What factors maintain it now? "Why has it not gotten better?"*
5) *What treatment or solution do you believe is needed?*
6) *Do you have any worries or fears about receiving treatment from a counselor?*"
7) *"Have there been negative experiences with counselors or mental health professionals in the past? If so, do you have concerns about engaging in treatment again? Do you have specific concerns?"*

Sometimes it is easiest to gain a description about a desired future by asking: "*What would be happening now in your family, if this problem were not present?*" (edi biraami bhaena bhane, tapaaiko pariwaar aajha bholi kusari pharak hola?) The answer to this question is often a description of a desired future.

Note for the counselor:

- *Praise/reward any effort a family member is making*
- *Talk openly with the family when one of the family members is not following the rules/guidelines, as described above. For example: mother and father are having a conflict with each other during the session and thereby ignoring all the rules. A possible consequence could be to stop this quarreling and ask them to be silent for a while and think about their behavior and the impact this may have on themselves and their child (time-out).*

b. Recognizing parental burden

Service providers should acknowledge and give attention to the difficulties the parents are experiencing in dealing with the problems in the family. Recognizing parental burden is important to demonstrate to the parents that the service provider is on 'their side'. This is an

attitude that the service provider needs to portray actively to the parents by focusing on their perceptions of distress.

Parental influence and parent-child relationships

a. *Parental influence and capacity*

The bases for continuing with the specific practice elements (see below) is discussing with parents about their perceived influence over their child's problems and/or the family situation. If parents do not perceive that they have any influence, this will need to be addressed by emphasizing and demonstrating the natural positive intentions and capacities of parents. Basically, all parents show examples of capacity and good intentions in their interactions, responses or wishes. Once that intention has been acknowledged, the service provider can start discussing about the effect that some influencing techniques may have on the child – setting the stage for the actual practice elements.

Strengths of the family:
It can also be helpful to ask about specific strengths of the family. It is possible to talk about the strengths of the family as a whole or the strengths of the family members individually (father, mother, child etc.), especially in relation to dealing with this problem or other major problems. In this way the focus will shift from problem-thinking to thinking about opportunities (how to solve/cope with these problems).

b. *Parent-child relationship (see also annex 1)*

A second 'prerequisite' before continuing is a discussion between service provider and parents about the parent-child relationship. A strained parent-child relationship should be discussed and addressed. If this relationship is found to be destructive, this may be a contra-indication to continue and further attention should be given to relational issues. Development of positive relations may be very different between settings and families, but typically include (i) providing time and attention to the child; (ii) talking or interacting with the child, and (iii) demonstrating affection.

If there is more clarity about the parental influence/capacity and the parent-child-relationship it is helpful to summarize and move toward a joint family plan of action. In this family plan of action the roles that everyone has in the change can be discussed. Techniques (as you can read further in this manual) can be useful in this joint family plan of action.

☆ Interventionists need to convey that parents are not to be blamed and that their support is very crucial in the intervention. Collaboration among child, parents and interventionist is crucial to instill change.

2.2 Parent Management Training [PMT] for Externalizing problems/ Disruptive behaviors

After the first sessions – covered under the name 'family intake' (see 2.1) – the service provider has:

- talked about and worked on the (pre)engagement
- found a way to address the family members
- discussed the rules and guidelines of the treatment
- found a mutual understanding of the problem
- recognized the parental burden
- an idea of the parental influence
- an idea of the parent-child relationship

- talked about the strengths of the family
- made a joint family plan of action

The next step is to make this joint family plan of action concrete with the techniques/tools this chapter is going to provide. Teachers and parents will learn how to respond to the child's behavioral problems. There are 5 different techniques they can use consequently help to change the behavioral problems of the child (see figure 6).

Figure 6: Disruptive behaviors practice elements

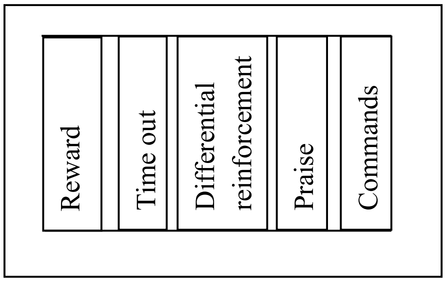

The service provider works with the parents on the several behavior modification techniques: (1) training of parents in a specific technique, (2) supervised implementation of the technique in the home setting, (3) evaluation of the effect of the technique.

1. Tangible rewards

The use of training of parents, teachers, or others involved in the social ecology of the child in the contingent administration of tangible rewards to promote desired behaviors. In a Nepali context, rather than involving tokens, charts, or record keeping, it more frequently involves direct (i.e., first order) reinforcers.

a. Selection of desired behaviors
b. Selection of reward system
   These may include; chocolate or chewing gum, books, clothes, verbal reinforcement (saying 'good job', 'I'm happy with you'), cooking a child their favorite food. Parents may also reward children by visiting places and physical affection such as hugs and kisses.
c. Using reward system *immediately* after desired behavior
d. Explanation of reason for reward (labeling)
e. Consistency

2. Time-out

The use of time-outs means the training of parents, teachers, or others involved in the social ecology of the child in the direct use of a technique involving removal of the youth from all reinforcement for a specified period of time following the performance of an identified, unwanted behavior. This can also involve the direct use of this strategy by the therapist. Methods of timeout may include; withholding of chocolates or foods or television viewing and locking children in the bathroom/separate room. An alternative strategy is employed in Nepal, where rather than separate a child in a room, parents tell the child, I am not happy with your behavior so I will not talk to you. The parents then do not speak with the child for a few minutes.

a. Instructions to child when starting time-out
   "I am not happy with your behavior, as a result I am giving you 'time out'"
b. Explanation to child for the specific reason for the time-out (labeling)

"You used bad language to me, which is not acceptable."

c. Determine period of time-out
   "I will now, not speak to you for 2 minutes."
d. What to do if the child does not respect the time-out?

3. Praise

The use of training of parents, teachers, or others involved in the social ecology[1] of the child in the administration of social rewards to promote desired behaviors. This can involve praise, encouragement, affection, or physical proximity.

a. Selection of desired behaviors
b. Praise compliments a child immediately after demonstrating positive targeted behavior.
   - Labeled praise is specific praise; ―You choose such pretty colors! ―You're being so careful with that pen!‖, ―I like it when you build quietly!‖
   - Unlabeled praise is non-specific: '*syabaas*', 'good job', 'I'm happy with you', 'very good', 'nice', 'my dear', 'my lovely child'.
c. Labeled praise is more effective because it:
   - lets the child know exactly what you like
   - increases the behavior that it describes
   - increases child's self-esteem[2]

4. Differential reinforcement

The use of differential reinforcement means the training of caretakers, teachers, or others involved in the social environment of the child to selectively ignore target behaviors and selectively attend to competing behaviors.

a. Selection of targeted behavior to ignore
- making too much noise or crying in an effort to get parents' attention

b. Selection of targeted behavior to promote
   For example children may give correct answers, use good communication, complete their homework, go to bed on time. Parents may respond with verbal encouragement, saying syabaas or non-verbal encouragement; patting them on the back, giving them the 'thumbs up' signal or giving them a hug. Parents may also provide verbal incentives; "If you study hard I will give you …bicycle, good notebook; if you do good in your studies and bring good marks we will take you to maternal home, we will cook your favorite food, we will buy you good clothes in festival, .If you stop playing I will provide chocolate/fruits , Going to fishing with parents, going to the shop.

5. Commands

The use of commands means the training for caretakers in how to give instructions or commands in such a manner as to increase the likelihood of child compliance.

a. Discussion of basic principles of giving instructions
   - Limited in number

---

[1] Social ecology refers to the nested arrangement of family, school, neighborhood, and community contexts in which children grow up.

[2] Eyberg, S. (2010). Parent Child Interaction Therapy. Integrity Checklists and Sessions Materials. University of Florida.

- o Simple to follow for the child (clarity of instruction)
- o Positive formulation of instruction
- o If child obeys give praise (see above)
- o If child disobeys give consequence (see above)

Example: If the child stays out too late at night, the parent may make a schedule together with the child, describing when the child is allowed to stay out late at night. The child and parent will decide together what the punishment will be if the child does not follow the schedule.

Example; if the child is not completing daily tasks, such as homework or not going to bed at the correct time, or eating meals, the parents may used commands; to instruct the child in what they need to do. The parent may also try to motivate the child by explaining the reality of the situation and providing choices. Eg: The parent may say, I have to go to work, you can eat your meal now, or if you choose not to eat your meal now, then you will not be able to eat later, as I will be at work and there will be no one to prepare a meal for you.

Problem solving and Monitoring
This approach is used if the problem is more complicated and requires multiple actions from the parents to resolve it.

a. *Alcohol/ substance abuse/gang fights*
   - Help the child understand the impact and consequences of their actions
   - Parents explain their disapproval, saying that they are "not happy" when children do these actions.
   - Check routine and daily activities, to what the child's daily activities are and whether changes need to be made.
   - Investigate the child's friendship circle and learn who their friends are.
   - Parent's network. Meet with parent's of child's friends to build relationships
   - Spend time with their child and give their child more attention. Eating meals together, particularly dinner, is an important part of this, to foster child-family unity and also to monitor the child's wellbeing.

b. *Truancy (non-school attendance)*
   - Find out the reasons behind this behavior
   - Investigate whether the child is doing more enjoyable activities outside school.
   - Asking the child each day specific questions regarding their school activities to gather information and monitor whether or not the child is attending school.

Monitoring and evaluation

As mentioned above the results of treatment should be monitored systematically. If the lower intensity care does not provide the expected gains (i.e. improvement in family functioning and reduction in the child's problems), stepping up to the next level of care is indicated. Stepping up requires a decision about patient progress based on judgments about 'significant health gain' or 'improvement'. Establishing such intervention impact might involve the use of personalized outcome indicators (or individualized expected outcomes). See annex 3.

- ➔ In case intervention gain is absent the family can be 'referred' to specialized services, if available.

Annex 1: Child-parent relationships[3]

A central tenant in improving children's psychosocial well-being through working with the parents is parent-child interaction patterns. Research has shown that a positive relationship between child and parents is an important protective factor for children's mental health and its development at large. A chief indicator for such positive relationship is *sensitive responsiveness,* which refers to the parents' ability for healthy communication. Working with parents on sensitive responsiveness has the following core principles; (a) focus on successful interaction and contact – this entails a shift in focus from problems and negative processes to a more positive and capacity oriented process; (b) Positive initiatives for contact by child - innate need and abilities for social contact (inter-subjectivity); (c) Wish for good contact by parents; (d) Existing capacities of parents and child within their natural setting; (e) Good interaction patterns between parents and child is the bases for the parents' role in raising children and for children's social-emotional development; (f) The use of direct feedback and indirect self-imposed modelling initially focus on awareness, increased sensitivity and secondly on actual change in behaviours.

[3] Based on Parent-Child Interaction Therapy (PCIT) concepts. PCIT is an evidenced-based treatment model with highly specified, step-by-step, live-coached sessions with both the parent/caregiver and the child. Parents learn skills through PCIT didactic sessions, and, using a transmitter and receiver system, the parent/caregiver is coached in specific skills as he or she interacts in specific play with the child.

Annex 2: Child rearing principles

Basic principles of positive child rearing (Triple-P) include: (1) providing a safe and stimulating environment for children, which should include physical and emotional safety; (2) providing positive attention and encouragement to support children in their endeavors to learn new things and skills; (3) providing a well-balanced discipline, which entails a consequent reaction towards negative or unwanted behaviors and clear rules about what is allowed and what is not; (4) providing the child with realistic, age sensitive, expectations and accepting children's mistakes; (5) taking care of oneself as parents.

Examples of topics for child rearing training:

1. Issues related to attachment and security and sensitive responsiveness
   - Parents should try to have a clear daily structure and routine
   - Clear communication of acceptance of the child
   - Communication that aims at sensitive responsiveness (see also below)

2. Issues related to basic behavior modification strategies
   - Increasing the parents' ability to identify when their child is experiencing problems
   - Use of attention, reward, punishment and instructions
   - It is important that the adults are consistent, predictable, fair, provide alternatives, and limit use of physical behavior change methods
   - Assisting and reinforcing the parents' existing positive child rearing strategies
   - It is important to be specific and realistic in formulating behavior change

3. Issues related to moving from dependence to independence
   - Trying to find a balance between letting the child experience/explore and setting limits/ clarity

4. Issues related to child and parental mastery
   - Learning of new behaviors to create a sense of control
   - Parents can be reassured that 'perfect parents' is not possible
   - Family/parents are the main teachers, children learn from their parents
   - Every child is unique, different children need different approaches
   - Child development & development tasks

Annex 3: Determining intervention gains (Personalized Outcome Indicators)

Personalized outcome indicators are to determine the clients most disturbing complaints that s/he would like to see changed as a result of the intervention. These items will be used as an outcome indicator before and after the intervention. Instructions; (a) List, in your own words, your most disturbing problems, which you expect the intervention to reduce? (Determine whether this is an IN: individual problem, FA: family problem, PE: peer problem, SC: school problem, NE: neighborhood problem, by circling the abbreviation in the last column.); (b) How much is this problem disturbing you in your day-to-day life? [Facilitator introduces 0-10 Subjective Unit of Distress Scale, with 0 indicating no distress at all, and 10 indicating too much distress to handle]; (c) Please complete before start of the intervention and after termination of the intervention. Rate each of the following problems according to how much it has bothered you during the past week, including today.

Problem/complaint	SUDS (0-10)	IN/ FA/ PE/ SC/ NE
[POI 1]		IN/ FA/ PE/ SC/ NE
[POI 2]		IN/ FA/ PE/ SC/ NE
[POI 3]		IN/ FA/ PE/ SC/ NE
[POI 4]		IN/ FA/ PE/ SC/ NE
[POI 5]		IN/ FA/ PE/ SC/ NE

IN: individual problem, FA: family problem, PE: peer problem, SC: school problem, NE: neighborhood problem

Annex 4: Creating mutual understanding

Any therapeutic process follows an assessment phase to create an in-depth understanding of the problems and reasons for the problems. Some of the issues that could be included in such assessment are:

1. Problem description and experience by parents
   - What is the problem, when does it occur, how often does it occur & who perceives it
   - What consequences (i.e. reactions) does the problem have for you, the child and the family
   - What would you like to see changed, what would the child like to be changed

2. What child factors play a role in causing and continuation of the problem?
   - Child's behaviors
   - Health problems
   - Child's character
   - Events that the child has gone through

3. What parental factors play a role in causing and continuation of the problem?
   - Problems in raising child
   - Marital tensions
   - Personal problems of parents
   - Social-economic reasons

4. What external factors play a role in causing and continuation of the problem?
   - Limited support system
   - Problems of extended family
